# Effects of Arbuscular Mycorrhizal Fungi and Clay Minerals on Soil Nitrogen and Phosphorus Availability and Microbial Community Responses

**DOI:** 10.3390/life16101625

**Published:** 2026-09-28

**Authors:** Zhirui Zhao, Jialong Luo, Zixuan Zhao, Guoxin Liu, Shijia Feng, Xiaogang Chen, Kui Cai, Zefeng Song

**Affiliations:** 1 College of Water Resources and Environment, Institute of Resources and Environmental, Hebei GEO University, Shijiazhuang 050031, China; zhiruizh@163.com (Z.Z.); luo596816276@163.com (J.L.); 18713183263@163.com (Z.Z.); lgx257076051@163.com (G.L.); 15844242102@163.com (S.F.); 18303383930@163.com (X.C.); kcai@hgu.edu.cn (K.C.); 2 Hebei Province Key Laboratory of Sustainable Use and Development of Water Resources, Shijiazhuang 050031, China

**Keywords:** AMF, clay minerals, microbial community, soil nutrients, bacteria

## Abstract

Efficient management of soil nitrogen and phosphorus availability is important for soil fertility and crop productivity, but the field responses of rhizosphere microbial communities to arbuscular mycorrhizal fungi (AMF) and clay-mineral amendments remain unclear. A wheat-field experiment compared four treatments: an untreated control (A), bentonite (B; 39 kg ${\mathrm{plot}}^{-1}$), sepiolite (C; 39 kg ${\mathrm{plot}}^{-1}$), and AMF inoculation (E; 2.25 kg inoculum ${\mathrm{plot}}^{-1}$; 120 spores $g^{-1}$). Each treatment comprised six 4 × 7.5 m plots. Soil physicochemical properties were evaluated using all six plots, whereas rhizosphere microbial sequencing used three plots per treatment (plots 2, 4, and 6; *n* = 3). Treatment E showed higher mean alkali-hydrolyzable nitrogen (101.50 vs. 93.33 mg ${\mathrm{kg}}^{-1}$) and available phosphorus (56.00 vs. 20.98 mg ${\mathrm{kg}}^{-1}$) than the control, whereas treatment B showed a 32.8% higher mean available potassium. None of the bacterial or AMF alpha-diversity indices differed significantly among treatments. Taxonomic summaries showed descriptive variation among treatments, whereas principal component analysis (PCA) and analysis of similarities (ANOSIM) did not detect significant treatment-level separation of either the bacterial or AMF communities. Shotgun metagenomic annotation, rather than 16S-based functional inference, further showed descriptive differences in nitrogen- and phosphorus-related gene-abundance profiles and functional potential; Pseudomonadota and Actinomycetota accounted for substantial shares of several nutrient-cycling gene categories, while pstB showed comparatively pronounced numerical variation among treatments. These metagenomic profiles represent genetic functional potential and do not demonstrate transcript abundance or metabolic activity. Overall, AMF inoculation and clay-mineral amendments were associated with descriptive differences in nutrient availability, taxonomic profiles, and metagenomic functional potential in alkaline wheat farmland, but significant treatment-level separation of the overall bacterial and AMF communities was not detected.

## 1. Introduction

Soil nitrogen (N) and phosphorus (P) availability is closely associated with soil fertility, crop productivity, and the functioning of rhizosphere microbial communities. In agricultural soils, particularly under alkaline conditions, nutrient availability depends not only on the total nutrient pool but also on biological transformation, mineral adsorption and desorption, and plant–microbe interactions. Therefore, management practices that improve nutrient acquisition while maintaining a favorable rhizosphere microbial environment are important for sustainable crop production.

Arbuscular mycorrhizal fungi (AMF) are obligate plant symbionts that associate with the roots of many crop species, including wheat. After colonizing roots, AMF develop extensive extraradical hyphal networks that extend beyond the nutrient-depletion zone around roots and increase the volume of soil explored by the plant. This mycorrhizal pathway is particularly important for the acquisition of relatively immobile nutrients such as P [1,2], while AMF can also participate directly and indirectly in soil N acquisition and cycling [3]. AMF have also been considered important biological agents for improving plant establishment and soil functioning in degraded ecosystems [4]. Field evidence further supports these functions in wheat. A meta-analysis of 38 field studies showed that AMF inoculation increased wheat biomass and the uptake of P, N, and Zn, and increased grain yield by approximately 20% on average, although the magnitude of the response depended strongly on soil properties, including pH, available P, total N, organic matter, and soil texture [5]. In addition to direct nutrient transfer, AMF can modify the rhizosphere environment and interactions among soil microorganisms, suggesting that their agronomic effects should be evaluated not only through plant or soil nutrient responses but also through changes in microbial community structure and nutrient-cycling functions.

Controlled-condition studies complement this field evidence. In a compartmented-pot experiment, AMF accounted for more than half of phosphorus uptake by wheat even when colonization did not produce a positive growth response [6]. Greenhouse and growth-chamber experiments with *Funneliformis mosseae* showed increased wheat biomass and grain yield together with confirmed root colonization [7]. In non-sterile agricultural soil under glasshouse conditions, a commercial *Rhizophagus irregularis* inoculum increased AMF root colonization across several wheat cultivars, although the resulting nutrient-acquisition benefits were variable [8]. These controlled studies demonstrate that AMF inoculants can influence wheat nutrition, growth, and root colonization, while also showing that inoculant efficacy depends strongly on the host and environmental context. This context dependence supports the need for field-scale evaluation.

Clay minerals provide a different pathway for regulating the soil nutrient environment. Bentonite is mainly composed of swelling smectite minerals such as montmorillonite and has a high capacity for water retention, cation exchange, and nutrient adsorption. Previous field studies have demonstrated that bentonite amendment can influence both soil fertility and microbial processes. Zhang et al. reported that bentonite increased soil water and nutrient retention and maize productivity in sandy soil and caused clear shifts in soil microbial composition and functional guilds, including groups associated with N cycling [9]. In a five-year field experiment in northern China, Mi et al. further showed that bentonite application increased soil organic C, total N and P, and microbial biomass C, N, and P, demonstrating that its effects extend beyond physical improvement of soil and can influence nutrient pools and microbial activity [10].

Sepiolite differs substantially from bentonite in mineral structure. It is a fibrous hydrated magnesium silicate characterized by a porous structure and strong adsorption capacity. Clay minerals have been widely investigated for the remediation of heavy-metal-polluted agricultural soils because their adsorption and ion-exchange properties can reduce the mobility and bioavailability of contaminants [11]. More specifically, agricultural studies of sepiolite have largely focused on the immobilization of potentially toxic elements, especially Cd and Pb, and have shown that sepiolite can modify soil physicochemical properties and consequently influence plant growth and soil processes [12]. Evidence also indicates that sepiolite can affect soil microbial communities. For example, Qin et al. used high-throughput sequencing to investigate sepiolite-amended red soil and found changes in microbial diversity and community composition after sepiolite application [13]. These findings indicate that clay minerals can function not only as physicochemical adsorbents but also as modifiers of microbial habitats. However, the microbial effects reported for bentonite and sepiolite have largely been examined in sandy soils, semi-arid soils, or contaminated soils, and the responses observed in these systems cannot be directly extrapolated to alkaline wheat farmland.

Despite substantial research on AMF and clay minerals individually, several questions remain unresolved. Previous AMF studies have primarily emphasized plant nutrient uptake, colonization, growth, and yield, whereas bentonite studies have mainly focused on soil water retention, physical properties, and nutrient preservation, and much of the sepiolite literature has concentrated on heavy-metal immobilization. Direct field-scale comparisons of AMF inoculation, bentonite, and sepiolite under the same agricultural conditions remain limited. In particular, it is still unclear whether these biologically and mineralogically distinct amendments regulate available N and P through similar or different changes in bacterial and AMF communities. Information is also limited regarding whether bentonite and sepiolite, because of their contrasting layered and fibrous structures, exert different ecological filtering effects on native AMF communities and on microbial functions associated with N and P cycling.

To address these gaps, a field experiment was conducted in wheat soil using four separately applied treatments: an untreated control, bentonite, sepiolite, and an AMF inoculum. The objectives were to (i) determine how the three amendments affect soil physicochemical properties and available N and P; (ii) characterize variation among treatments in bacterial and AMF community diversity and composition; and (iii) compare microbial functional potential associated with N and P cycling. The functional analyses were based on shotgun metagenomic sequencing rather than 16S-based functional prediction and therefore represent gene-abundance patterns and functional potential rather than transcript-level activity or measured metabolic rates. We hypothesized that AMF inoculation would increase available N and P and would be associated with variation in bacterial community composition through mycorrhizal nutrient acquisition and rhizosphere microbial interactions. We further predicted that bentonite and sepiolite would primarily modify nutrient retention and microbial habitats and, because of their contrasting mineral structures, would be associated with different response patterns in bacterial community composition and native AMF richness and diversity. The novelty of this study lies in the side-by-side field comparison of a biological amendment (AMF inoculum) and two mineral amendments with contrasting structures (bentonite and sepiolite), while integrating soil nutrient availability, bacterial and AMF community responses, and shotgun-metagenomic N- and P-related functional profiles within the same field framework.

## 2. Materials and Methods

### 2.1. Experimental Materials

The AMF used in this study was an indigenous isolate obtained from soil collected at the study site. Spores were recovered by wet-sieving and decanting followed by sucrose centrifugation. The recovered spores were transferred to Petri dishes and examined under a stereomicroscope (SMZ745T, Nikon, Tokyo, Japan). Based on spore size, color, shape, wall layers, and subtending-hypha characters, compared with the VA mycorrhizal identification manual and INVAM descriptions, the isolate was morphologically assigned to *Septoglomus constrictum* (syn. *Glomus constrictum*). The same isolate was screened and described in Meng Xiangyuan’s 2022 master’s thesis [14]; that earlier study used wheat-host propagation and measured colonization in a separate controlled experiment, not in the present field plots. Spores showing consistent morphological characteristics were selected and propagated using wheat as the host plant. After propagation, the wheat shoots were removed, and the colonized roots and rhizosphere substrate were cut and homogenized under aseptic conditions to prepare a composite AMF inoculum containing spores, hyphae, colonized root fragments, and growth substrate. The inoculum contained 120 spores $g^{-1}$. Because isolate-specific molecular barcoding was not performed, the species-level assignment reported here is based on morphological identification. Field root colonization by the inoculated isolate was not quantified in this field experiment, and no inoculant-specific molecular marker or post-application barcoding assay was used. Therefore, the study evaluates the effects associated with application of this AMF inoculum but does not directly demonstrate field establishment, dominance, or displacement of indigenous AMF by the inoculated isolate.

Bentonite was purchased from Zhangjiakou Xuanhua District Lishi Bentonite Processing Co., Ltd. (Zhangjiakou, China), and sepiolite was purchased from Yixian Sepiolite Development Co., Ltd. (Baoding, China).

The commercial bentonite and sepiolite applied in the field were obtained from the suppliers named above. An archived major-oxide composition summary from the actual applied batches, with material headings corrected against their oxide profiles, records for bentonite: ${\mathrm{SiO}}_{2}$ 71.58%, ${\mathrm{Al}}_{2}O_{3}$ 14.79%, MgO 5.35%, and CaO 3.05%; and for sepiolite: ${\mathrm{SiO}}_{2}$ 33.79%, MgO 18.50%, CaO 26.70%, and ${\mathrm{Al}}_{2}O_{3}$ 3.73%. These figures are the retained batch summary rather than independently repeated analyses in this study. X-ray diffraction/mineral-phase composition, mineral purity, cation-exchange capacity, specific surface area, particle-size distribution, and complete analytical-method documentation were not available; therefore, literature-based mineral properties are not presented as measurements of these batches.

### 2.2. Study Area and Experimental Design

The field experiment was conducted in a wheat field in Qingyuan District, Baoding City, Hebei Province, China, near the border with Anxin County, under conventional local field management conditions. The original field trial comprised 36 plots arranged as a 6 × 6 Latin square with six treatments, with each treatment occurring once in each row and once in each column. Thus, treatment allocation followed a spatially structured Latin-square design rather than an unrestricted completely randomized design. The present study focused on four treatments from that field trial: an untreated control (A1–A6), bentonite treatment (B1–B6), sepiolite treatment (C1–C6), and AMF inoculation treatment (E1–E6), comprising 24 plots in total. Each plot measured 4 m × 7.5 m ($30\;m^{2}$), and each treatment contained six independent replicate plots. One-meter-wide buffer strips were established around the plots and along the field boundary to reduce interference between adjacent plots. The six rows and six columns represented the two spatial directions of the field layout and provided row–column control of spatial heterogeneity. The available field records do not document the specific randomization or permutation procedure used to generate the Latin-square allocation. The parent experiment also contained a bentonite + sepiolite treatment (D) and an AMF + bentonite + sepiolite treatment (F).

The experiment was conducted in situ in the existing wheat field. An archived photograph dated 28 March 2024, confirmed by the authors to depict this trial, records 38.820642° N, 115.682649° E for a photographed untreated A plot. This is a plot location, not a surveyed center coordinate or boundary of the whole field. Formal soil taxonomic classification and a complete set of pretreatment soil physicochemical baseline measurements were not retained. The measurements in Table 1 are wheat-maturity observations for all treatment groups; the untreated A plots received no amendment but are not pretreatment baseline soil.

For treatment B, 39 kg of bentonite was applied to each $30\;m^{2}$ plot, corresponding to 1.3 kg $m^{-2}$. For treatment C, 39 kg of sepiolite was applied to each $30\;m^{2}$ plot, corresponding to 1.3 kg $m^{-2}$. Treatment E received 2.25 kg of AMF inoculum per plot, with an inoculum density of 120 spores $g^{-1}$.

The archived field records available for this study do not specify the exact amendment-application date or frequency, whether bentonite and sepiolite were surface-applied or incorporated into the soil, or the depth of incorporation. These details were therefore not reconstructed from the separate root-box experiment or from general agronomic practice.

A single wheat cultivar was used across all plots, and wheat was sown at the locally conventional sowing rate with the same sowing rate maintained among plots; however, the cultivar name and exact sowing rate were not retained in the available field records. The field plot was considered the experimental unit, with six independent biological replicates for each treatment. The experiment was conducted under local conventional management and ambient field conditions until wheat maturity. Specific fertilizer products, application rates and timing, and irrigation dates or amounts were not documented in the records available for this study, so no unverified management details are reported.

### 2.3. Sample Collection

At wheat maturity in mid-June 2024, soil and plant samples were collected from the field experiment. For physicochemical analyses, soil was sampled from the same 24 field plots comprising treatments A, B, C, and E, with six independent replicate plots per treatment.

For physicochemical analyses, five soil subsamples were collected independently from the 0–20 cm layer below the soil surface within each plot using the five-point sampling method. The five subsamples from each plot were thoroughly homogenized to obtain one representative plot-level composite soil sample. Thus, a total of 24 composite soil samples were obtained for physicochemical measurements. The samples were placed in sampling bags, kept in a cooled sampling box during transportation, and transferred to the laboratory.

Microbial samples were collected separately from the rhizosphere of representative wheat plants. Within the complete 36-plot field experiment, three wheat plants were randomly selected in each plot, and one of these three plants was then randomly chosen as the representative plant for that plot. Rhizosphere soil associated with the root system of the selected representative plant was collected at wheat maturity in mid-June 2024 and stored at −80 °C. For the four treatments analyzed in this study, six rhizosphere samples were therefore available per treatment. After field sampling and before DNA extraction and library preparation, the sequencing subset was designated using the predefined even-numbered-plot rule: A2, A4, and A6; B2, B4, and B6; C2, C4, and C6; and E2, E4, and E6. Thus, plant selection within plots was randomized, whereas the final choice of three field plots per treatment for sequencing followed a systematic plot-number rule. All 12 sequenced plots were also included in the physicochemical dataset because physicochemical measurements were performed on all six plots per treatment. However, the within-plot sample types differed: physicochemical measurements used five-point composite samples from the 0–20 cm soil layer, whereas sequencing used representative-plant rhizosphere soil from the same field plots.

### 2.4. Soil Physicochemical Analysis

Soil samples were air-dried in a cool and well-ventilated environment, after which visible gravel, plant roots, and other debris were removed. The dried soil was ground and passed through a 2 mm sieve before physicochemical analysis. Soil pH was determined in a soil–water suspension at a ratio of 1:2.5 (*w*/*v*). Briefly, 10 g of soil was mixed with 25 mL of deionized water and allowed to equilibrate for 30 min before measurement using a portable multiparameter meter (Multi 3630, WTW, Weilheim, Germany).

Other soil physicochemical properties were determined according to the methods described in Soil Agrochemical Analysis [15]. Soil organic carbon (SOC) was measured using the potassium dichromate volumetric method. Total nitrogen (TN) was determined by potassium dichromate–sulfuric acid digestion, whereas alkali-hydrolyzable nitrogen (AN) was measured using the alkaline hydrolysis diffusion method. Total phosphorus (TP) was determined by sulfuric acid–perchloric acid digestion, and total potassium (TK) was measured using NaOH fusion followed by flame photometry. Available phosphorus (AP) was extracted with sodium bicarbonate and determined using the molybdenum–antimony colorimetric method, while available potassium (AK) was extracted with ammonium acetate and quantified by flame photometry.

### 2.5. DNA Extraction and High-Throughput Sequencing

For the sequencing-based analyses, a two-stage sampling and selection procedure was used. First, at wheat maturity in mid-June 2024, three wheat plants were randomly selected within each field plot, and one of these three plants was randomly chosen as the representative plant. Rhizosphere soil from the root system of this representative plant was collected and stored at −80 °C. Second, after field sampling and before DNA extraction and library preparation, the representative rhizosphere samples from the even-numbered plots were designated for sequencing: A2, A4, and A6 for the control; B2, B4, and B6 for the bentonite treatment; C2, C4, and C6 for the sepiolite treatment; and E2, E4, and E6 for the AMF treatment. Therefore, plant selection within plots was random, whereas the final plot subset used for sequencing followed a predefined systematic rule. Each treatment contained three independent plot-level biological replicates (*n* = 3). Physicochemical analyses were conducted on all six plots per treatment, so every sequenced plot was also represented in the physicochemical dataset; nevertheless, physicochemical and sequencing measurements were made on different within-plot soil samples (five-point composite topsoil versus representative-plant rhizosphere soil, respectively). Approximately 0.3 g of fresh rhizosphere soil from each selected sample was used for total microbial DNA extraction using the FastDNA Spin Kit for Soil (MP Biomedicals, Irvine, CA, USA) according to the manufacturer’s instructions. The reported sequencing replicates were independent field-plot biological replicates rather than technical replicates.

For bacterial community analysis, the V3–V4 region of the bacterial 16S rRNA gene was amplified using primers 338F (5′-ACTCCTACGGGAGGCAGCAG-3′) and 806R (5′-GGACTACHVGGGTWTCTAAT-3′). For AMF-targeted 18S rRNA analysis, the archived provider PCR report documents a two-round nested amplification: first AML1F–AML2R (55 °C, 32 cycles), then AMV4-5NF (5′-AAGCTCGTAGTTGAATTTCG-3′)–AMDGR (5′-CCCAACTATCCCTATTAATCAT-3′) (55 °C, 25 cycles). The first primer pair follows the AMF 18S primer design described by Lee et al. [16]; the provider report confirms the pair names but does not itself list their oligonucleotide sequences. The second reaction used a 20 μL FastPfu system. PCR amplification used TransStart FastPfu DNA Polymerase (AP221-02, TransGen Biotech Co., Ltd., Beijing, China) on an ABI GeneAmp 9700 thermocycler (Applied Biosystems, Foster City, CA, USA). A preliminary amplification check was recorded for representative samples; the cycle numbers above are those reported for the archived AMF nested-PCR experiment. The bacterial PCR cycle count is not established by the retained report. Each DNA sample was amplified in triplicate. The three PCR products from the same biological sample were pooled, checked by 2% agarose gel electrophoresis, purified using an AxyPrep DNA Gel Extraction Kit (Axygen Biosciences, Union City, CA, USA), eluted in Tris-HCl, and rechecked by 2% agarose gel electrophoresis. These PCR replicates were technical replicates and were not treated as independent observations.

The pooled amplicons were quantified using a QuantiFluor-ST fluorescence quantification system (Promega, Madison, WI, USA), and sequencing libraries were prepared using the TruSeq DNA Sample Prep Kit (Illumina, San Diego, CA, USA). Bacterial 16S rRNA and AMF 18S rRNA amplicon libraries were sequenced on the Illumina MiSeq platform using paired-end 2 × 300 bp sequencing by Majorbio Bio-Pharm Technology Co., Ltd. (Shanghai, China). For the 12 field-plot samples analyzed in this study, the optimized bacterial 16S dataset contained 53,434–74,127 sequences per sample (mean, 61,232), with Q30 values of 96.40–97.02%; the optimized AMF 18S dataset contained 79,090–98,383 sequences per sample (mean, 88,037), with Q30 values of 97.89–98.40%. The AMF-targeted 18S amplicon sequencing was used to characterize AMF community composition in field rhizosphere soil; it was not an isolate-specific tracking assay and therefore cannot by itself confirm persistence or root colonization of *Septoglomus constrictum* after field application.

Shotgun metagenomic sequencing was additionally performed on the same three biological replicates per treatment to characterize microbial functional genes involved in nitrogen and phosphorus metabolism. After genomic-DNA quality assessment by 1% agarose gel electrophoresis, DNA was fragmented to an average size of approximately 350 bp using a Covaris M220 instrument, and metagenomic libraries were constructed using the NEXTFLEX Rapid DNA-Seq Kit. Libraries were sequenced on the Illumina NovaSeq 6000 platform using paired-end 2 × 150 bp sequencing. Across the 12 A, B, C, and E samples analyzed here, quality-controlled metagenomic depth ranged from 65,987,562 to 88,476,878 clean reads per sample (mean, 75,451,013). The sample-to-SRA-run mapping for the 12 sequenced plots is given in Supplementary Table S1, submitted as a separate file. The retained completion report provides the depth range and mean stated above, but a verified per-sample clean-read/quality table was not available for this revision.

### 2.6. Bioinformatics and Statistical Analysis

Raw paired-end amplicon reads were demultiplexed and quality-filtered using fastp v0.23.4 [17], followed by paired-end merging using FLASH v1.2.11 [18]. Project-specific quality-filtering parameters were taken from the sequencing completion report: bases with quality scores below 20 were trimmed using a 50-bp sliding window; reads shorter than 50 bp after filtering or containing ambiguous bases (N) were discarded. Paired-end reads were merged with a minimum overlap of 10 bp and a maximum mismatch ratio of 0.2. Barcode mismatches were not permitted, whereas a maximum of two primer mismatches was allowed. The resulting high-quality sequences were processed within the Majorbio amplicon-analysis workflow [19] to obtain amplicon sequence variants (ASVs), which were used for taxonomic composition and diversity analyses. Alpha diversity was evaluated using the ACE, Chao1, Sobs, Shannon, Simpson, and Good’s coverage indices. ACE, Chao1, and Sobs were interpreted as richness estimators, whereas Shannon and Simpson were used to characterize diversity and/or dominance patterns; Good’s coverage was used as an estimate of sampling completeness. Shared and treatment-specific ASV counts were summarized using petal/Venn-style diagrams. Genus-level heatmaps were used as descriptive visualizations of treatment-associated taxonomic patterns. The heatmap displays 20 bacterial taxa and 5 AMF taxa selected for visualization; taxa not displayed in the heatmap were not removed from the underlying ASV dataset or from upstream community-level analyses. The heatmaps were therefore interpreted as descriptive summaries rather than formal differential-abundance tests. Between-sample community variation was visualized on the Majorbio Cloud platform [19] using principal component analysis (PCA) based on the resulting community-abundance profiles. The bacterial PCA was conducted at the ASV level, whereas the AMF PCA was conducted at the genus level, consistent with the retained analysis outputs. The first two principal components were plotted, and treatment-level separation was evaluated using analysis of similarities (ANOSIM) with 999 permutations. An ANOSIM *p*-value below 0.05 was considered evidence of significant group separation; R values near or below zero indicate that between-group dissimilarity did not exceed within-group dissimilarity. Soil physicochemical measurements were obtained from six independent field plots per treatment (*n* = 6) and organized in Microsoft Excel. The archived manuscript dataset available for the present revision contains treatment-level minimum–maximum summaries rather than the six plot-level values required to calculate valid standard deviations, omnibus test statistics, and pairwise *p*-values. Accordingly, Table 1 and Section 3.1 report these physicochemical data descriptively, and no inferential significance is assigned to the numerical treatment differences. Amplicon alpha-diversity analyses were performed on the Majorbio Cloud platform [19] using three independent field-plot samples per treatment (*n* = 3). Overall differences among treatments A, B, C, and E were assessed for each alpha-diversity index using the Kruskal–Wallis H test. Because only three independent biological replicates were available per treatment, distributional assumptions could not be assessed robustly and formal normality testing would have very low power at this sample size. The Kruskal–Wallis test was therefore used as a conservative rank-based omnibus test that does not require a normality assumption. No transformation was applied to the alpha-diversity indices. To account for testing six alpha-diversity indices within each community type, raw *p*-values were adjusted separately for bacterial and AMF communities using the Benjamini–Hochberg false-discovery-rate (FDR) procedure. An FDR-adjusted q-value < 0.05 was considered statistically significant. Because none of the omnibus Kruskal–Wallis tests remained significant after FDR adjustment, no post-hoc pairwise comparisons were performed. Because only three independent field plots per treatment were sequenced, statistical power was limited, particularly for small-to-moderate treatment effects. Consequently, non-significant results were interpreted as insufficient evidence of a treatment difference rather than evidence of equivalence, and taxonomic and functional patterns derived from these three replicates were treated as exploratory or descriptive unless supported by formal statistical tests.

For metagenomic functional analysis, raw reads were preprocessed with fastp v0.23.0 [17]. Quality-controlled reads were assembled using MEGAHIT v1.2.9 [20], and open reading frames were predicted using Prodigal v2.6.3 [21]. Contigs ≥ 500 bp were retained for downstream gene prediction, and predicted genes with nucleotide lengths ≥ 100 bp were retained. A non-redundant gene catalog was constructed using CD-HIT v4.6.1 [22] with 90% sequence identity and 90% coverage. Gene abundance was quantified using Salmon v1.10.3 [23] with a 95% identity threshold. Taxonomic annotation was performed using DIAMOND v2.0.13 [24] against the NCBI NR database [25] with an E-value cutoff of 1 × $10^{-5}$. Functional annotation was performed against the KEGG database [26] using DIAMOND with the same E-value cutoff. Following the platform workflow, abundances of genes assigned to the same KEGG gene, KO, EC, or pathway category were summed to obtain category-level abundance profiles. These profiles are presented as relative contribution percentages within the displayed functional summaries; in the stacked-bar taxonomic-contribution summary, each bar sums to 100%. An archived platform-settings screenshot for the nitrogen-cycle annotation specifies reads-per-kilobase-per-million (RPKM) abundance estimation and within-group median summaries; the displayed category profiles were then converted to relative-contribution percentages. Whether exactly the same settings generated every nitrogen and phosphorus functional-profile panel cannot be established from that screenshot alone, and project-specific groupwise p-values were not available for the displayed functional profiles. Accordingly, these functional comparisons are treated as descriptive and exploratory, and no statistical significance is claimed for them. Importantly, the functional results in Section 3.3 were derived from shotgun metagenomic gene annotation and were not inferred from the 16S rRNA amplicon data.

## 3. Results and Discussion

### 3.1. Soil Physicochemical Properties and Nutrient Availability

The effects of bentonite, sepiolite, and AMF inoculation on soil physicochemical properties and nutrient availability were evaluated descriptively relative to the untreated control (Table 1). Mean total nitrogen was 1.84 g ${\mathrm{kg}}^{-1}$ in treatment A and 1.80 g ${\mathrm{kg}}^{-1}$ in treatment E. Mean alkali-hydrolyzable nitrogen was 93.33 mg ${\mathrm{kg}}^{-1}$ in A and 101.50 mg ${\mathrm{kg}}^{-1}$ in E, an 8.7% numerical increase. Mean available phosphorus was 20.98 mg ${\mathrm{kg}}^{-1}$ in A and 56.00 mg ${\mathrm{kg}}^{-1}$ in E, while mean soil pH was 8.04 and 7.84, respectively. Available potassium is reported separately for the mineral treatments rather than pooled: treatment B had a mean of 259.05 mg ${\mathrm{kg}}^{-1}$ with an observed range of 183.12–327.97 mg ${\mathrm{kg}}^{-1}$, whereas treatment C had an observed range of 175.00–257.88 mg ${\mathrm{kg}}^{-1}$. The control A had a mean available-potassium value of 195.07 mg ${\mathrm{kg}}^{-1}$, and treatment E had a mean of 261.18 mg ${\mathrm{kg}}^{-1}$. Because valid SDs and overall/pairwise test statistics cannot be reconstructed from minimum–maximum summaries alone, these physicochemical differences are interpreted as descriptive numerical patterns rather than statistically demonstrated treatment effects.

AMF can contribute substantially to plant P acquisition through the mycorrhizal pathway and can also participate in N acquisition and cycling [1,2,3]. Field and synthesis studies in wheat further indicate that AMF inoculation can influence nutrient acquisition and N-use efficiency, although the magnitude of the response depends on soil nutrient conditions and management practices [5,27]. AMF acquire mineral nutrients through extensive extraradical hyphae while receiving photosynthetically derived carbon from their host plants [28,29], and N taken up by AMF can be assimilated and transferred within the symbiosis [30]. More broadly, soil microorganisms and mycorrhizal associations play important roles in plant mineral nutrition and nutrient availability [31,32]. These established processes provide a physiological and ecological context for the higher available N and P observed under treatment E, although the present experiment did not directly quantify nutrient transfer through AMF hyphae. Phosphate availability is also strongly controlled by soil fixation, biological turnover, and plant–microbial interactions [33,34,35], which may explain why available P responded more strongly than total P.

The higher available K observed under bentonite treatment is consistent with the physicochemical properties of swelling clay minerals. Bentonite can improve soil physical conditions and nutrient retention, and field studies have reported changes in soil biochemical properties and microbial communities following bentonite amendment [9,10]. More broadly, clay minerals can modify soil chemical conditions and ion availability through adsorption and ion-exchange processes [11], while sepiolite has also been widely investigated for its strong sorption capacity and effects on soil properties [12]. The parent field experiment also contained combined treatments, including bentonite + sepiolite (D) and AMF + bentonite + sepiolite (F). However, the present manuscript intentionally focuses on A, B, C, and E to compare the individual responses associated with the untreated control, each clay mineral, and AMF inoculation under the same field setting. Because the parent trial was not a complete factorial design and did not include separate AMF + bentonite and AMF + sepiolite combinations, formal AMF × mineral interaction effects cannot be estimated cleanly from this treatment structure. Accordingly, synergistic effects of combined amendments are outside the scope of the present analysis and are not inferred from the single-amendment comparisons.

### 3.2. Bacterial and AMF Community Responses

To determine whether the observed nutrient responses were accompanied by changes in soil microbial communities, bacterial and AMF diversity and composition were subsequently compared among treatments. Soil amendments can influence microbial communities through changes in nutrient availability, water retention, pore structure, and microscale habitat properties [9,13,36,37]. AMF may additionally modify microbial assemblages through interactions with bacteria in the mycorrhizosphere and hyphosphere [38,39,40]. More generally, plant roots and their associated microbiomes are strongly linked through resource inputs and biotic interactions [31,41,42], while soil biodiversity and community composition can influence ecosystem functioning without necessarily changing in parallel [43].

#### 3.2.1. Alpha Diversity

For bacterial communities, treatment A showed the highest numerical values for the ACE, Chao1, and Sobs richness indices, whereas treatments B and C tended to have lower richness values and treatment E showed intermediate values. However, none of the six bacterial alpha-diversity indices differed significantly among treatments according to the Kruskal–Wallis H test: ACE (*p* = 0.1834), Chao1 (*p* = 0.1834), Sobs (*p* = 0.1473), Shannon (*p* = 0.516), Simpson (*p* = 0.6793), and Good’s coverage (*p* = 0.2699). After Benjamini–Hochberg FDR correction, all adjusted q-values remained > 0.05 (q = 0.3668, 0.3668, 0.3668, 0.6192, 0.6793, and 0.4049, respectively). Therefore, the observed richness differences should be regarded as numerical trends rather than statistically supported treatment effects. Amendment-dependent changes in microbial richness and community composition have been reported in soils receiving AMF, biochar, or bentonite [9,36], but the nonsignificant alpha-diversity tests in this small sequencing subset do not establish stability or equivalence among treatments (Figure 1).

A similar pattern was observed for AMF communities. Treatment A showed the highest numerical richness, whereas treatment C had the highest Shannon index (3.08). Treatment E showed a lower Shannon index (2.50) and a higher Simpson index (0.15). Nevertheless, none of the AMF alpha-diversity indices differed significantly among treatments: ACE (*p* = 0.3331), Chao1 (*p* = 0.3297), Sobs (*p* = 0.2796), Shannon (*p* = 0.09867), Simpson (*p* = 0.1009), and Good’s coverage (*p* = 0.2402). After Benjamini–Hochberg FDR correction, all adjusted q-values remained > 0.05 (q = 0.3331, 0.3331, 0.3331, 0.3027, 0.3027, and 0.3331, respectively). Soil P availability can influence AMF spore germination and hyphal growth [44], while AMF communities are also shaped by host–fungus interactions and the surrounding microbial environment [29,38,39,40]. These mechanisms provide plausible ecological context for the observed numerical patterns, but they do not substitute for statistical evidence of treatment effects (Figure 2).

Taken together, the alpha-diversity results provide no statistically supported evidence that the three amendments altered overall bacterial or AMF richness/diversity under the present sequencing design after FDR correction. Because *n* = 3 per treatment provides limited power, these non-significant results should not be interpreted as proof of equivalence. Between-sample community patterns were therefore examined separately using PCA and ANOSIM, as reported below.

#### 3.2.2. Between-Sample Community Variation

The bacterial PCA at the ASV level showed that PC1 and PC2 explained 9.74% and 9.69% of the variation, respectively (19.43% combined). Samples from the four treatments overlapped broadly, and ANOSIM did not detect significant group separation (R = −0.1728, *p* = 0.929). For the AMF community at the genus level, PC1 and PC2 explained 38.74% and 24.23% of the variation, respectively (62.97% combined). Although the ordination displayed dispersion among individual samples, the treatment ellipses overlapped and ANOSIM again did not detect significant separation (R = −0.1142, *p* = 0.708). Thus, the PCA patterns primarily reflect within-treatment variation and are not interpreted as evidence of treatment-level whole-community separation (Figure 3).

#### 3.2.3. ASV Distribution and Taxonomic Profiles

Although α diversity remained statistically similar among treatments, ASV distributions showed descriptive variation in observed community membership. In bacterial communities, 1076 ASVs were shared by all four treatments, whereas 4952, 4391, 4511, and 4593 treatment-specific ASVs were observed in A, B, C, and E, respectively. For AMF communities, 29 ASVs were shared among all four treatments, while the numbers of treatment-specific ASVs were 99, 90, 92, and 83, respectively. These pooled counts document shared and treatment-specific detections but do not by themselves establish a statistically significant treatment effect on overall community composition. Changes in soil physical structure and nutrient distribution can alter microbial niches at the microscale [37], while AMF-associated hyphae can provide distinct habitats and carbon inputs for bacterial assemblages [28,38,39,45] (Figure 4).

The bacterial heatmap further showed descriptive variation in dominant taxa among treatments. RB41, members of Blastocatellaceae, *Terrimonas*, and *Nitrospira* showed relatively high values in the untreated control, whereas *Bacillus* was highest under bentonite treatment, *Arthrobacter* under sepiolite treatment, and *Steroidobacter* under AMF inoculation. Some Acidobacteriota are associated with soil-adapted and relatively oligotrophic lifestyles [46,47], and *Vicinamibacter* represents a lineage within Acidobacteria subdivision 6 [48]. *Nitrospira* comprises important nitrite-oxidizing microorganisms [49]. *Terrimonas* is a soil-associated bacterial genus [50], *Steroidobacter* includes denitrifying and steroid-degrading representatives [51], *Acidibacter* includes acidophilic gammaproteobacteria [52], and *Rubrobacter* species are known for pronounced environmental stress tolerance [53]. These known traits provide ecological context for the observed distributions but do not demonstrate that the same physiological functions were expressed in the present soil samples. Previous studies have shown that bentonite and sepiolite amendments can alter soil microbial community composition [9,13]. In the present dataset, however, the heatmap patterns are interpreted as descriptive relative-abundance variation rather than statistically demonstrated enrichment, suppression, or whole-community separation.

The AMF heatmap likewise indicated descriptive variation in taxonomic profiles. *Sclerocystis* showed its highest observed abundance under treatment E, whereas *Claroideoglomus* followed the order A > E > C > B. Interactions between AMF and rhizosphere bacteria can affect nutrient utilization and microbial community organization [38,40], and AMF extraradical hyphae can recruit specific hyphosphere bacterial communities involved in P turnover [54]. Soil P status can also influence AMF spore germination and hyphal development [44]. Together with the broader ecological role of mycorrhizal fungi [29], these studies provide context for the numerical AMF patterns observed here. However, the higher abundance of *Sclerocystis* under treatment E cannot be regarded as direct evidence of successful colonization by the inoculated strain because the inoculum was morphologically identified as *Septoglomus constrictum* and isolate-specific molecular tracking was not performed (Figure 5). Accordingly, the observed AMF profile is treated as descriptive variation associated with AMF inoculum application; establishment of the inoculated isolate and competitive exclusion of indigenous AMF remain possible explanations rather than demonstrated mechanisms.

Collectively, the ASV-distribution and heatmap panels show taxon-level descriptive variation, whereas PCA and ANOSIM provide no evidence of significant treatment-level separation. These visual patterns are therefore not presented as statistically demonstrated whole-community shifts, particularly given the small sequencing sample size (*n* = 3 per treatment).

### 3.3. Shotgun Metagenomic Nitrogen- and Phosphorus-Related Functional Profiles

To further link microbial community responses with nutrient-cycling processes, shotgun metagenomic gene-abundance profiles associated with nitrogen and phosphorus cycling and their principal taxonomic contributors were examined descriptively among treatments. Because project-specific inferential test results were not available for these functional profiles, treatment-associated differences in this section are reported as numerical patterns rather than statistically significant effects.

In the nitrogen-related metagenomic profile, glutamine synthetase-associated annotations, including *glnA/GLUL* and K01915, represented the largest component, accounting for approximately 22.4% of the displayed annotated abundance. Glutamine synthetase is a central enzyme in microbial ammonium assimilation and provides an important route for incorporating inorganic N into cellular organic N [55]. The similar relative abundance of this functional category among treatments is consistent with comparatively stable ammonium-assimilation functional potential across treatments. This observation reflects gene-content potential and should not be interpreted as *glnA* expression, glutamine synthetase activity, or a measured ammonium-assimilation rate. Other N-related gene annotations, including *gltB*, *nirK*, *narG/narZ/nxrA*, *nasC/nasA*, *ureC*, and *norB*, showed smaller numerical variations among treatments. Denitrification involves the sequential reduction of nitrogen oxides and is widely distributed among soil microorganisms [56], whereas assimilatory nitrate reduction enables microorganisms to convert nitrate into reduced N for biosynthesis [57]. These pathway descriptions provide biological context for the annotated gene categories but do not demonstrate in situ denitrification or nitrate-assimilation activity. AMF can also transfer recently fixed plant carbon to surrounding soil microorganisms through the mycorrhizal pathway [28,45], and N transfer within the AM symbiosis has been demonstrated experimentally [30]. These processes provide possible ecological context for descriptive variation in the taxonomic sources of N-related metagenomic annotations even when the overall proportions of major functional categories remain relatively stable (Figure 6).

Phosphorus-related metagenomic profiles included gene annotations associated with phosphate transport, organic and inorganic P transformation, P regulation, and polyphosphate metabolism. Among them, *pstB* (K02036) showed one of the larger numerical differences in relative contribution among treatments. PstB is the ATP-binding component of the high-affinity phosphate-specific transport system and provides energy for phosphate uptake [58]. The higher relative *pstB* contribution under treatment B is therefore interpreted as a difference in metagenomic phosphate-transport potential, not as increased *pstB* expression, ATPase activity, or a measured phosphate-uptake rate. Microorganisms can influence soil P availability through phosphate solubilization, organic P mineralization, high-affinity phosphate transport, and polyphosphate metabolism [34,35]. The Pho regulon coordinates bacterial responses to phosphate limitation and integrates phosphate sensing, transport, and regulation [59]. Polyphosphate metabolism is also an important microbial strategy for P storage and utilization [60]. Within Actinomycetota, phosphate acquisition, Pst transport, and phosphate-regulatory systems are closely integrated [61], and phosphate-solubilizing members of Actinomycetota have been isolated from phosphate-rich mineral environments [62]. AMF-associated hyphosphere microbiomes can further contribute to P turnover and organic P mobilization [54]. Accordingly, the observed P-related profiles are interpreted as descriptive differences in genetic functional potential and not as evidence of transcriptional regulation or metabolic activity (Figure 7).

Taxonomic contribution analysis assigned substantial shares of organic-N-metabolism- and denitrification-related gene annotations to Pseudomonadota and Actinomycetota, while Pseudomonadota also accounted for a relatively large share of annotations related to assimilatory nitrate reduction and N transport. These patterns concern annotated genetic potential and should not be interpreted as direct evidence of denitrification rates or pathway activity [56,57]. For P-related functions, Pseudomonadota and Actinomycetota accounted for substantial shares of annotations related to phosphate transport and polyphosphate metabolism, consistent with known bacterial P-transport, storage, and regulation mechanisms [34,35,58,59,60,61,62]. The broader role of soil microorganisms in plant mineral nutrition [32] and ecosystem multifunctionality [31,43] supports interpreting these patterns at the level of metagenomic functional potential rather than assigning an active mechanism to any one taxon (Figure 8).

Taken together, the results indicate descriptive treatment-associated patterns linking soil nutrient availability, taxonomic profiles, and nutrient-related metagenomic functional potential. AMF inoculation was associated mainly with higher available N and P, whereas bentonite showed a stronger numerical response in available K. ASV distributions and dominant-taxon profiles varied among treatments, but alpha-diversity tests and PCA/ANOSIM did not provide statistically significant evidence of treatment-level separation of the bacterial or AMF communities. At the metagenomic functional-potential level, dominant N-related gene categories remained comparatively stable, while several P-related categories and their taxonomic contributors showed greater numerical variation among treatments. Because these functional profiles were descriptive and were not independently validated by transcriptomics, targeted qPCR, enzyme assays, or process-rate measurements, they should be interpreted as treatment-associated differences in genetic functional potential rather than gene expression or metabolic activity. A further limitation is that all sequencing-based analyses were based on only three independent field plots per treatment. This limited replication reduces statistical power and increases uncertainty around small or moderate treatment effects; therefore, non-significant results should not be taken as evidence of equivalence, and sequencing-derived community and functional patterns should be regarded as preliminary until confirmed with larger field replication. Likewise, because root colonization and inoculant-specific persistence were not measured, the present field data do not support claims that the inoculated AMF established, became dominant, or displaced indigenous AMF.

## 4. Conclusions

This field study compared the individual effects of an arbuscular mycorrhizal fungi (AMF) inoculum, bentonite, and sepiolite on soil nutrient availability, bacterial and AMF communities, and nitrogen- and phosphorus-related metagenomic functional potential in alkaline wheat farmland. Treatment E showed higher mean alkali-hydrolyzable nitrogen and available phosphorus than the untreated control, whereas bentonite treatment showed a higher mean available-potassium level. Because the replicate-level physicochemical dataset required for valid variance estimates and inferential tests was not recoverable for the present revision, these soil differences are interpreted as descriptive numerical patterns rather than statistically demonstrated treatment effects. Bacterial and AMF alpha-diversity indices did not differ significantly among treatments after FDR correction. ASV distributions and dominant-taxon profiles showed descriptive variation, but PCA and ANOSIM did not detect significant treatment-level separation of either community. Given the limited sequencing replication (*n* = 3 per treatment), the taxonomic patterns should therefore be regarded as exploratory rather than evidence of statistically demonstrated whole-community shifts. Shotgun metagenomic analysis indicated comparatively stable major nitrogen-related functional categories together with more pronounced numerical variation in some phosphorus-related categories, including pstB-associated annotations; Pseudomonadota and Actinomycetota were important contributors to several nutrient-cycling functional categories. These metagenomic results represent genetic functional potential rather than gene expression, enzyme activity, or measured process rates. In addition, root colonization by the inoculated AMF isolate and inoculant-specific molecular tracking were not measured, so successful establishment, dominance, or displacement of indigenous AMF cannot be inferred. Overall, the results provide a field-scale descriptive comparison of nutrient and rhizosphere microbial response patterns associated with individual AMF and clay-mineral amendments, while also defining priorities for future work, including larger sequencing replication, direct AMF-colonization measurements, process-level functional validation, and factorial evaluation of combined AMF–mineral treatments.

## Figures and Tables

**Figure 1:**
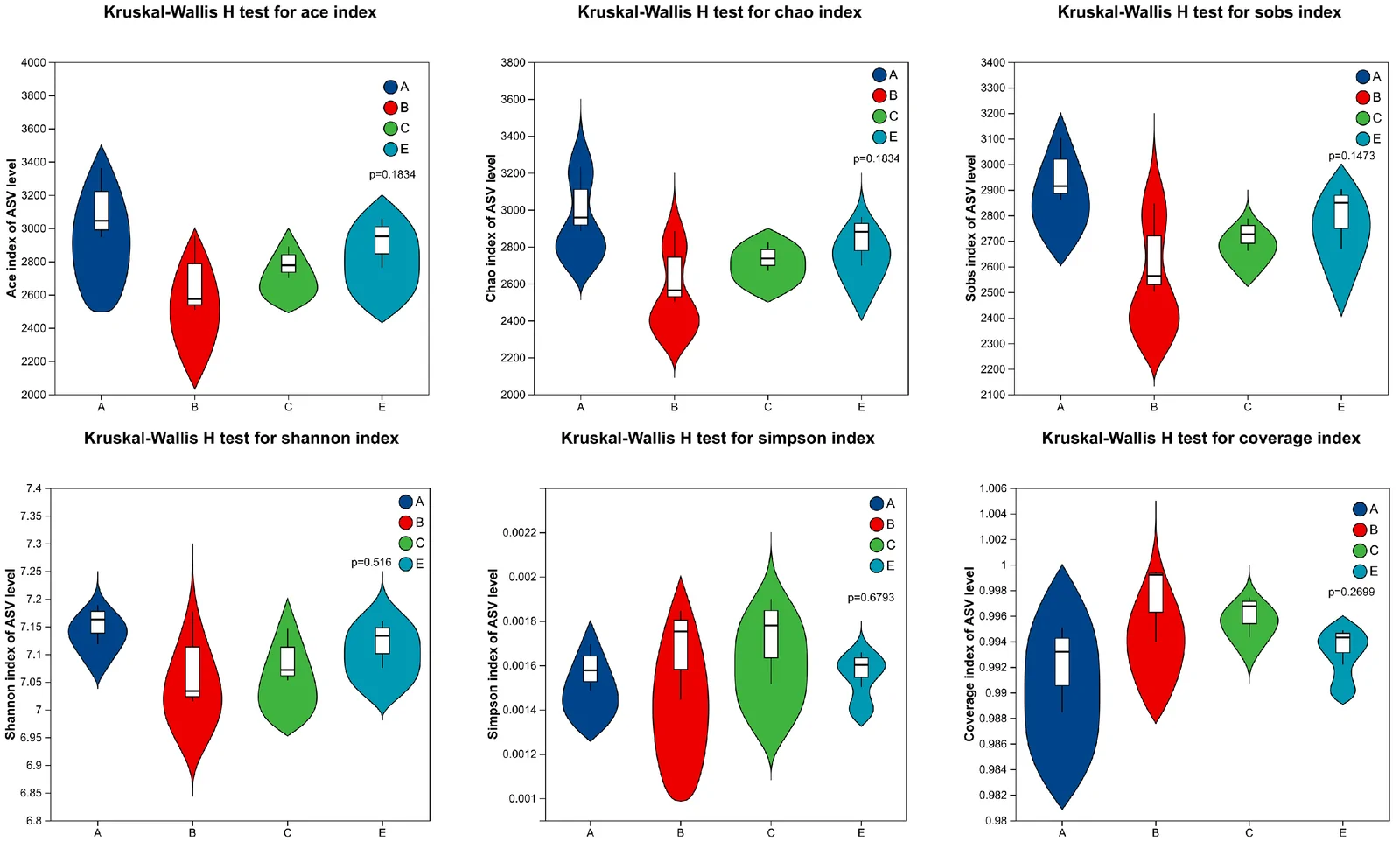
Alpha-diversity indices of bacterial communities under the four field treatments. Violin plots show ACE, Chao1, Sobs, Shannon, Simpson, and Good’s coverage indices (left to right, top to bottom). Colors denote treatments: A, untreated control (dark blue); B, bentonite (red); C, sepiolite (green); and E, AMF inoculant (teal). White boxes show the interquartile range, center lines show the median, and whiskers show the data range. Displayed *p*-values are raw omnibus values from the Kruskal-Wallis H test (*n* = 3); statistical inference was based on Benjamini–Hochberg FDR-adjusted q-values, all of which were >0.05.

**Figure 2:**
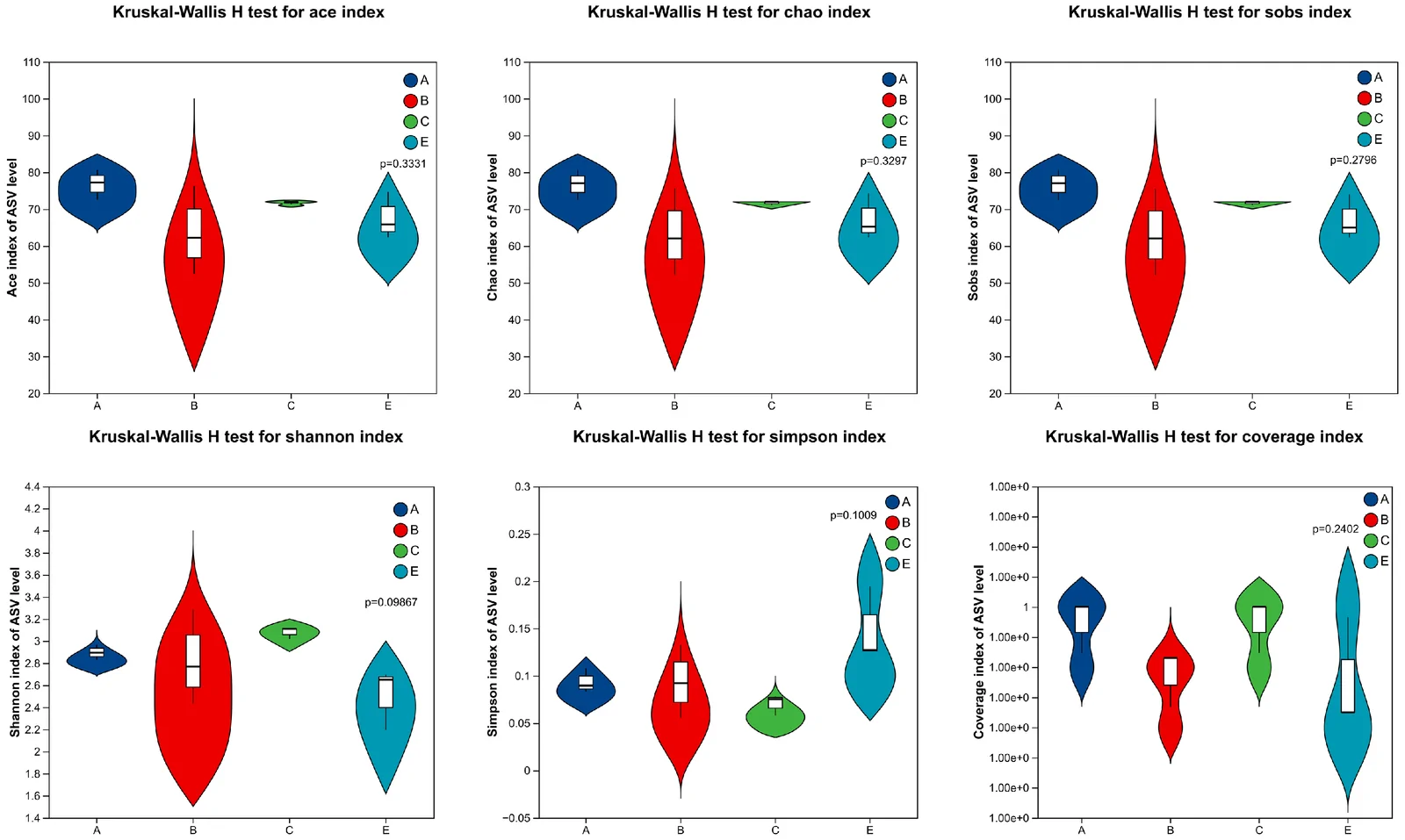
Alpha-diversity indices of AMF-targeted 18S rRNA communities under the four field treatments. Violin plots show ACE, Chao1, Sobs, Shannon, Simpson, and Good’s coverage indices (left to right, top to bottom). Colors denote treatments: A, untreated control (dark blue); B, bentonite (red); C, sepiolite (green); and E, AMF inoculant (teal). White boxes show the interquartile range, center lines show the median, and whiskers show the data range. Displayed *p*-values are raw omnibus values from the Kruskal-Wallis H test (*n* = 3); statistical inference was based on Benjamini–Hochberg FDR-adjusted q-values, all of which were >0.05.

**Figure 3:**
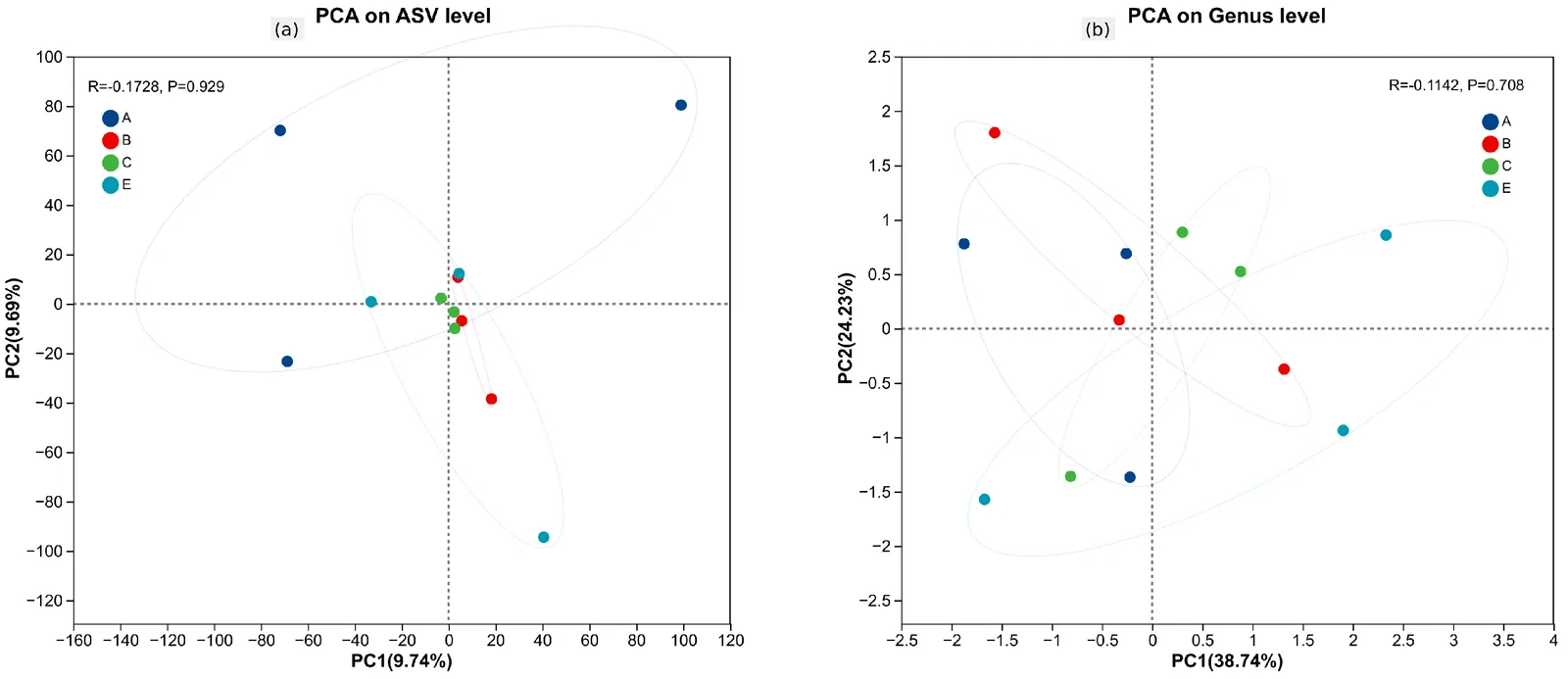
Principal component analysis (PCA) of between-sample community variation under treatments A, B, C, and E for (**a**) bacterial communities at the ASV level and (**b**) AMF-targeted communities at the genus level. Each point represents one independent field-plot sample (*n* = 3 per treatment); colors denote A, untreated control; B, bentonite; C, sepiolite; and E, AMF inoculant. Ellipses show the dispersion of samples within each treatment as displayed in the retained analysis output. Percentages on the axes indicate the variation explained by each principal component. ANOSIM statistics shown in the panels were calculated using 999 permutations. Neither bacterial nor AMF communities showed significant treatment-level separation (*p* > 0.05).

**Figure 4:**
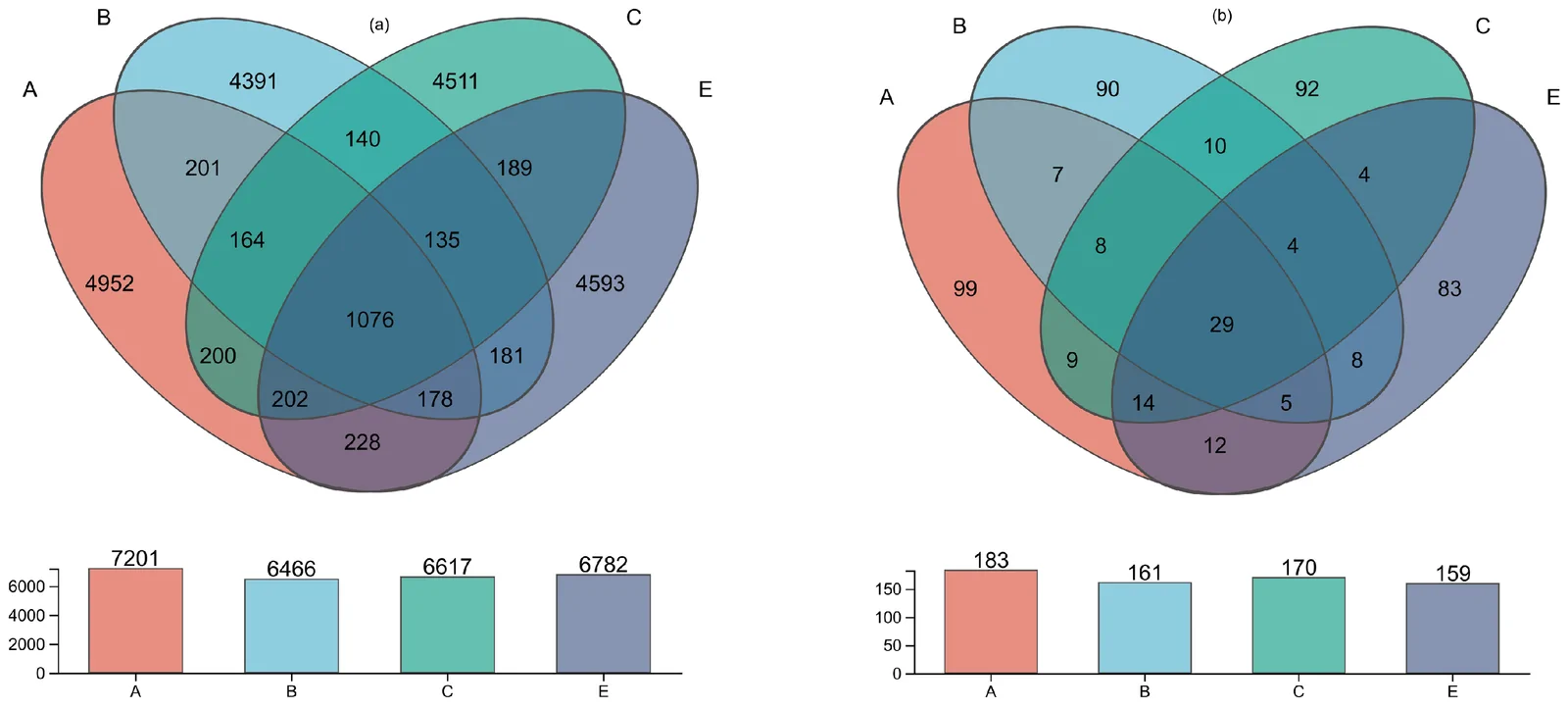
Shared and unique amplicon sequence variants (ASVs) among treatments A, B, C, and E for (**a**) bacterial and (**b**) AMF communities. Petal colors identify the treatments: A, untreated control (salmon); B, bentonite (light blue); C, sepiolite (green); and E, AMF inoculant (blue-gray). Numbers in overlapping regions indicate shared ASVs, numbers in non-overlapping regions indicate treatment-specific ASVs, and the bars below each diagram show the total ASV count per treatment. These pooled counts are descriptive and do not constitute a test of whole-community separation.

**Figure 5:**
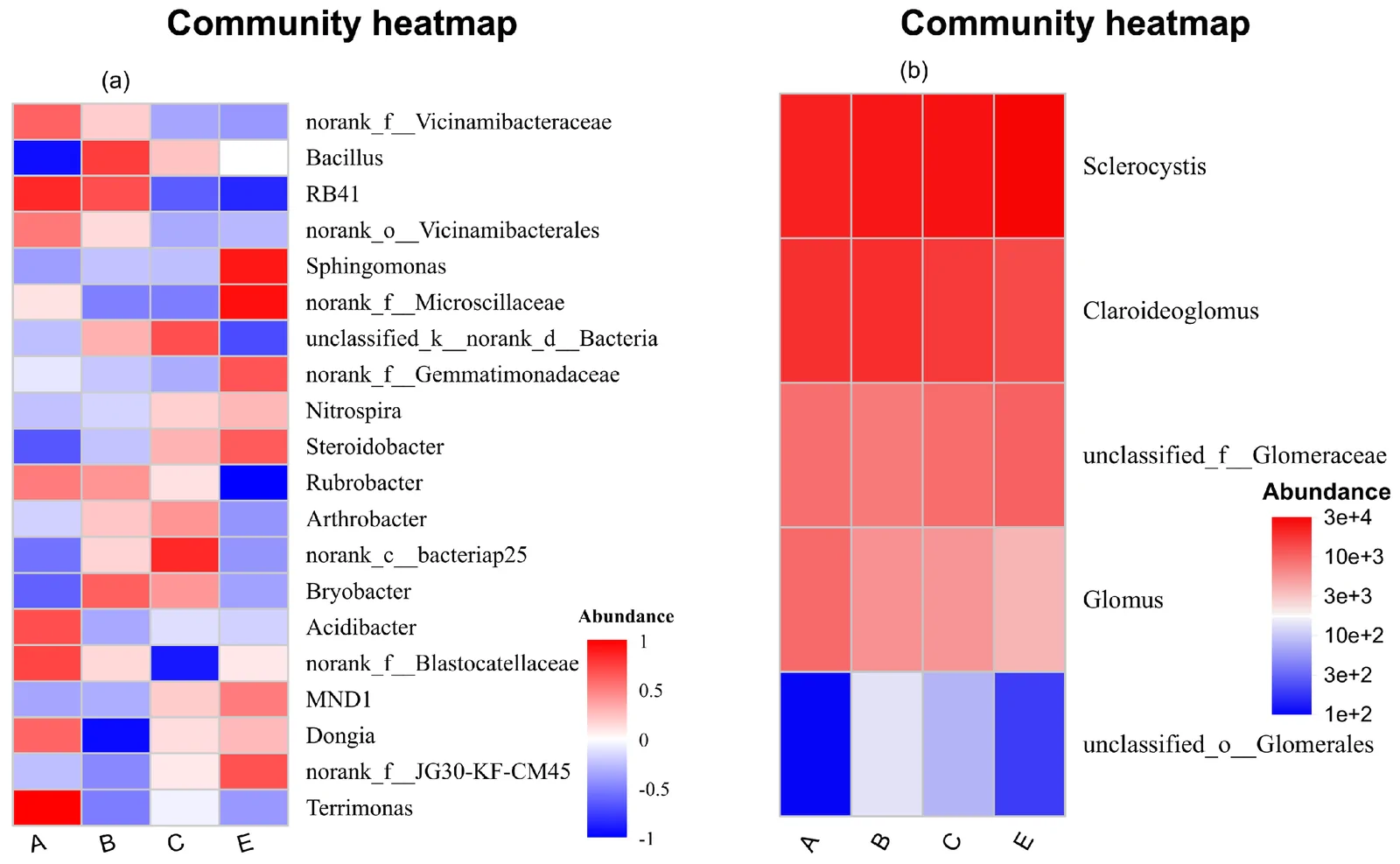
Heatmaps of selected dominant taxa in (**a**) bacterial and (**b**) AMF-targeted 18S rRNA communities under treatments A, B, C, and E. Panel (**a**) displays 20 bacterial taxa and panel (**b**) displays 5 AMF taxa selected for visualization. Taxa not shown were retained in the underlying ASV dataset and in upstream community-level analyses; their omission from the heatmap was for readability only. Columns represent treatments and rows represent taxa. Red indicates higher abundance, white indicates intermediate abundance, and blue indicates lower abundance according to the color keys shown; panel (**a**) displays standardized abundance values from −1 to 1, whereas panel (**b**) displays the abundance scale shown in the legend. These heatmaps are descriptive visualizations and do not constitute formal differential-abundance tests or evidence of whole-community separation. A, untreated control; B, bentonite; C, sepiolite; E, AMF inoculant.

**Figure 6:**
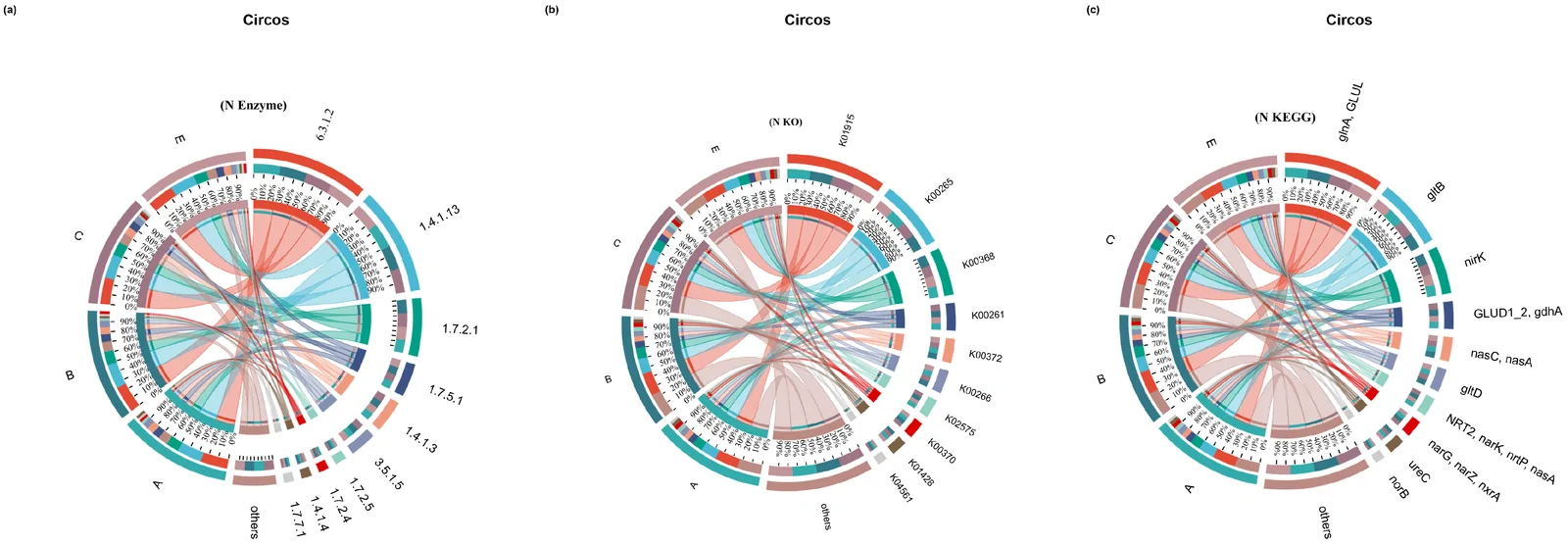
Nitrogen-metabolism functional profiles under treatments A, B, C, and E based on shotgun metagenomic (**a**) enzyme commission annotations, (**b**) KEGG Orthology (KO) entries, and (**c**) KEGG gene annotations. In each chord diagram, the left sectors represent treatments (A, untreated control, turquoise; B, bentonite, dark teal; C, sepiolite, gray-purple; E, AMF inoculant, rose), and the right sectors represent individual nitrogen-related annotations distinguished by color. Ribbons connect treatments with functional annotations; ribbon width and sector size indicate relative contributions, and tick marks show percentages within sectors. These values represent metagenomic gene-abundance potential and should not be interpreted as gene expression or metabolic-rate measurements.

**Figure 7:**
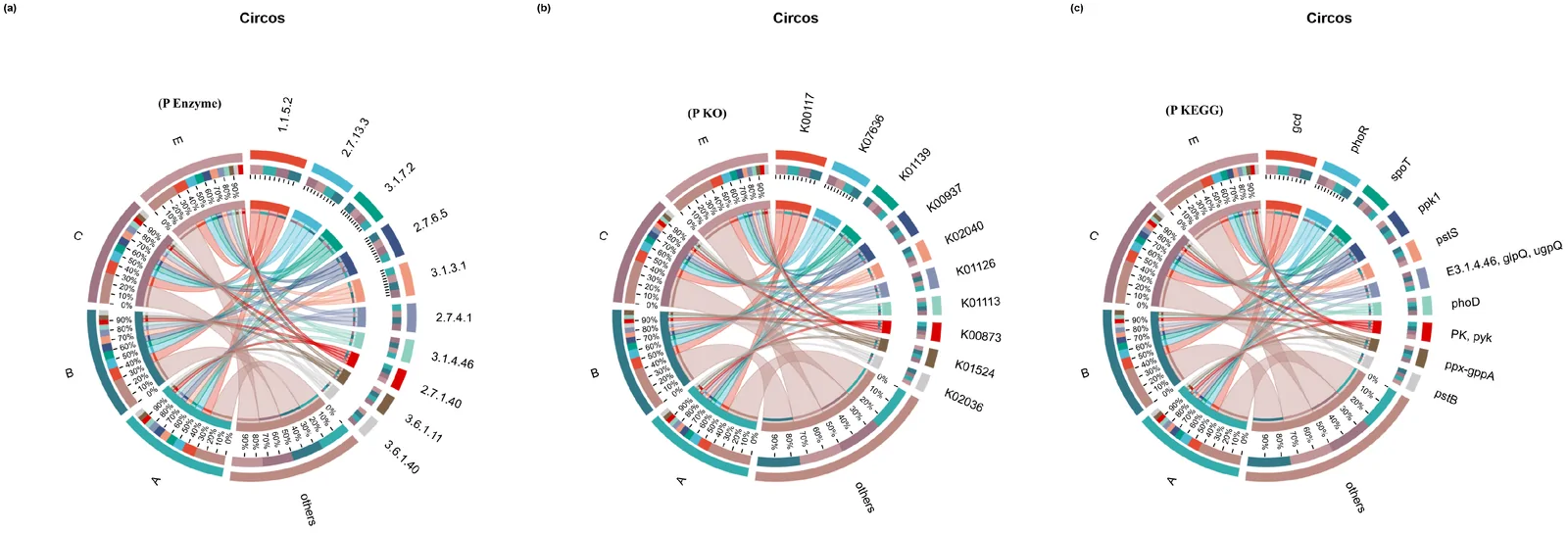
Phosphorus-metabolism functional profiles under treatments A, B, C, and E based on shotgun metagenomic (**a**) enzyme commission annotations, (**b**) KEGG Orthology (KO) entries, and (**c**) KEGG gene annotations. In each chord diagram, the left sectors represent treatments (A, untreated control, turquoise; B, bentonite, dark teal; C, sepiolite, gray-purple; E, AMF inoculant, rose), and the right sectors represent individual phosphorus-related annotations distinguished by color. Ribbons connect treatments with functional annotations; ribbon width and sector size indicate relative contributions, and tick marks show percentages within sectors. These values represent metagenomic gene-abundance potential and should not be interpreted as gene expression or metabolic-rate measurements.

**Figure 8:**
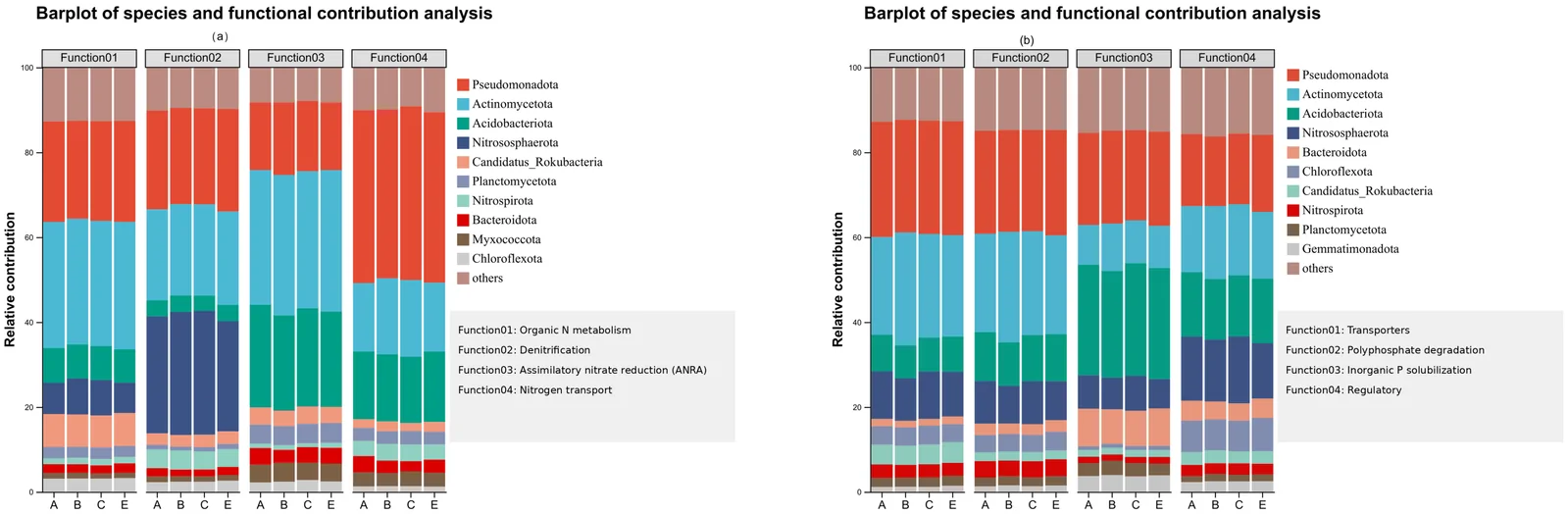
Relative contributions of bacterial phyla to shotgun metagenomic (**a**) nitrogen- and (**b**) phosphorus-metabolism gene categories under treatments A, B, C, and E. Each stacked bar sums to 100%, and colors identify bacterial phyla as listed in the legend; ‘others’ combines the remaining phyla. In panel (**a**), Function01, Function02, Function03, and Function04 denote gene categories related to organic nitrogen metabolism, denitrification, assimilatory nitrate reduction, and nitrogen transport, respectively. In panel (**b**), Function01, Function02, Function03, and Function04 denote gene categories related to phosphorus transport, polyphosphate degradation, inorganic phosphorus solubilization, and phosphorus regulation, respectively. A, untreated control; B, bentonite; C, sepiolite; E, AMF inoculant. The relative contributions describe annotated genetic potential, not measured pathway activity.

**Table 1:** Observed ranges of soil physicochemical properties under different treatments (*n* = 6 independent field plots per treatment).

Treatment	pH	SOC (g ${\mathrm{kg}}^{-1}$)	TN (g ${\mathrm{kg}}^{-1}$)	TP (g ${\mathrm{kg}}^{-1}$)	TK (g ${\mathrm{kg}}^{-1}$)	AK (mg ${\mathrm{kg}}^{-1}$)	AN (mg ${\mathrm{kg}}^{-1}$)	AP (mg ${\mathrm{kg}}^{-1}$)
A	7.99–8.07	19.99–28.16	1.66–1.95	0.87–1.26	16.87–19.23	155.07–233.71	77.00–105.00	16.87–24.13
B	7.99–8.01	19.70–25.94	1.67–1.86	0.87–1.05	19.06–20.23	183.12–327.97	84.00–105.00	12.05–22.86
C	7.88–8.03	19.30–21.11	1.68–1.76	1.02–1.26	17.95–19.19	175.00–257.88	94.50–98.00	13.99–51.66
E	7.62–7.95	18.62–22.57	1.70–1.88	0.71–1.32	15.41–19.27	212.05–317.66	98.00–105.00	24.47–76.83

Note: Values are observed minimum–maximum ranges across six independent field plots per treatment. The range summaries do not permit reconstruction of standard deviations, omnibus test statistics, or pairwise *p*-values; therefore, no significance notation is assigned in this table. B and C are separate treatments and are not pooled for available-potassium reporting.

## Data Availability

All bacterial 16S rRNA, AMF-targeted 18S rRNA, and shotgun-metagenomic raw sequencing records for this study are assigned to the single NCBI BioProject PRJNA1518313 (submission SUB16430201). NCBI Manage Data lists 36 BioSamples and 90 SRA objects as processed. Supplementary Table S1 maps the 12 field plots analyzed here to their corresponding SRR accessions for each of the three library types. At the time of revision, the project remained under embargo with a scheduled release date of 24 August 2028. The NCBI Reviewer link can show submission metadata during peer review but does not grant access to raw reads; the release date will need to be advanced when public data access is required for publication.

## Supplementary Materials

The following supporting information can be downloaded at: https://www.mdpi.com/article/10.3390/life16101625/s1, Table S1: Mapping of the 12 field plots analyzed in this study to their corresponding SRA run (SRR) accessions for bacterial 16S rRNA, AMF-targeted 18S rRNA, and shotgun-metagenomic libraries.
