# Supplementary material for: Effects of Arbuscular Mycorrhizal Fungi and Clay Minerals on Soil Nitrogen and Phosphorus Availability and Microbial Community Responses
Source: Life (Basel). 2026 Sep 28;16(10):1625. doi: 10.3390/life16101625 (PMC13706055; doi:10.3390/life16101625)
Supplement: Supplementary file 1 [file life-16-01625-s001.zip › life-4557964-supplementary.pdf]

Supplementary Table S1

Manuscript ID: life-4557964

NCBI SRA run accessions for the 12 A, B, C, and E field plots analyzed in this manuscript. All three library types are registered under BioProject PRJNA1518313 (submission SUB16430201).

**Plot**	**Bacterial 16S Illumina MiSeq**	**AMF 18S Illumina MiSeq**	**Shotgun WGS NovaSeq 6000**
A2	SRR40341398	SRR40341358	SRR40341409
A4	SRR40341395	SRR40341356	SRR40341408
A6	SRR40341393	SRR40341354	SRR40341397
B2	SRR40341391	SRR40341351	SRR40341386
B4	SRR40341389	SRR40341349	SRR40341375
B6	SRR40341387	SRR40341347	SRR40341364
C2	SRR40341384	SRR40341345	SRR40341353
C4	SRR40341382	SRR40341343	SRR40341342
C6	SRR40341380	SRR40341340	SRR40341331
E2	SRR40341371	SRR40341332	SRR40341405
E4	SRR40341369	SRR40341329	SRR40341404
E6	SRR40341367	SRR40341327	SRR40341403

Note: These SRR accessions were assigned in the NCBI processed submission receipt. The BioProject is currently under embargo; the scheduled release date shown in NCBI Manage Data at revision was 24 August 2028.
